# New Insights into Control of Arbovirus Replication and Spread by Insect RNA Interference Pathways

**DOI:** 10.3390/insects3020511

**Published:** 2012-05-29

**Authors:** Claire L. Donald, Alain Kohl, Esther Schnettler

**Affiliations:** MRC-University of Glasgow Centre for Virus Research, 8 Church Street, Glasgow G11 5JR, Scotland, UK; E-Mails: c.donald.1@research.gla.ac.uk (C.L.D.); alain.kohl@glasgow.ac.uk (A.K.)

**Keywords:** arbovirus, mosquito, small RNAs, RNA interference, RNAi, RNA silencing, antiviral immunity

## Abstract

Arthropod-borne (arbo) viruses are transmitted by vectors, such as mosquitoes, to susceptible vertebrates. Recent research has shown that arbovirus replication and spread in mosquitoes is not passively tolerated but induces host responses to control these pathogens. Small RNA-mediated host responses are key players among these antiviral immune strategies. Studies into one such small RNA-mediated antiviral response, the exogenous RNA interference (RNAi) pathway, have generated a wealth of information on the functions of this mechanism and the enzymes which mediate antiviral activities. However, other small RNA-mediated host responses may also be involved in modulating antiviral activity. The aim of this review is to summarize recent research into the nature of small RNA-mediated antiviral responses in mosquitoes and to discuss future directions for this relatively new area of research.

## 1. Introduction

Arthropod-borne viruses, or arboviruses, are a considerable threat to human and animal health in many parts of the world. These pathogens are transmitted by arthropod vectors such as mosquitoes, midges and ticks to susceptible vertebrates, and are often prominent examples of emerging and re-emerging viruses. Many arboviruses relevant to human and veterinary medicine are categorized into four virus families, the *Flaviviridae*, *Bunyaviridae*, *Togaviridae* (*Alphavirus* genus) and *Reoviridae*; this includes examples such as dengue virus (DENV), Rift Valley fever virus (RVFV), chikungunya virus (CHIKV) and bluetongue virus (BTV) [1]. CHIKV, DENV and BTV are becoming increasingly relevant in Europe [1,2,3,4,5,6,7] and the emergence of potential new arboviruses, such as the Schmallenberg virus (SBV; an orthobunyavirus infecting cattle and sheep and most likely transmitted by midges) in North/Western Europe, highlights the threat associated with new arthropod-borne pathogens [8]. The arthropod vector plays a crucial role within the arbovirus transmission cycle. The emergence of arboviruses is linked to the availability of suitable vectors or the introduction of new vectors (for example *Aedes albopictus* in the European Mediterranean basin), as well as the type of arbovirus. Many other factors such as geography/climate, trade, transport and socioeconomic factors are also relevant to the emergence of arboviruses and future risk assessments [1,9,10]. Understanding the biology of arthropod vectors is a key factor in understanding arbovirus replication and transmission. New discoveries, especially in the field of vector immune responses to arbovirus infection, have an increasing impact on our understanding of this virus/host interaction. As genetic modification of mosquito immunity has now been used to produce *Plasmodium*-resistant mosquitoes [11,12], further work on antiviral immunity in arbovirus vectors may also find new angles to extend these approaches. Recent research has enhanced our understanding of this antiviral response. This review summarizes our current understanding of the RNA interference (RNAi) pathways, which are key mechanisms in controlling arbovirus replication.

**Figure 1:**
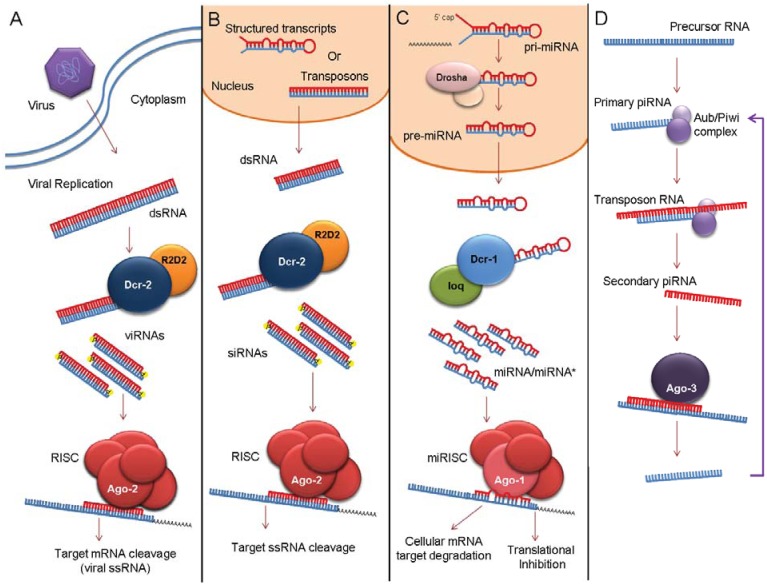
Schematic representation of the exogenous siRNA (**A**), endogenous siRNA (**B**), microRNA (miRNA) (**C**), and PIWI-interacting RNA (piRNA) (**D**) pathways present in insects; presented for *Drosophila melanogaster*. dsRNA, double stranded RNA; Dcr, Dicer; RISC, RNA-induced silencing complex; Ago, Argonaut; viRNA, viral specific small interfering RNA; siRNA, small interfering RNA; loq, loquacious; ssRNA, single stranded RNA.

A number of studies in recent years have shown that following infection, mosquitoes and mosquito derived cell lines mount an innate antiviral response both *in vivo *and *in vitro *(reviewed in [13]). To date, RNAi is considered to be the most significant innate antiviral immune response in insects [14] although the Toll, IMD and JAK/STAT pathways have also been shown to be important [13,14]. RNAi as an antiviral response is evolutionarily conserved in many organisms, including plants, fungi and insects. Most information regarding the RNAi pathways and the biology of small RNAs in invertebrates comes from studies in the model organism *Drosophila melanogaster* which has proven to be a good model for other insects [15]. So far, three major types of small RNA molecules have been identified; small interfering RNA (siRNA), microRNA (miRNA) and PIWI-interacting RNA (piRNA). These molecules have diverged roles in different cellular processes and virus-host interactions (Figure 1A–D). The siRNA pathway can be divided into endogenous and exogenous branches, depending on the source of the long double stranded (ds)RNA inducer molecule. 

## 2. Exogenous (antiviral) siRNA Pathway

Long dsRNA is frequently associated with virus infection [13]. These molecules are recognized in infected arthropod cells as pathogen-associated molecular patterns (PAMPs) and are detected by dicer RNaseIII endoribonucleases [15,16] (Figure 1A). In the model insect organism, *D. melanogaster*, this dsRNA substrate is cleaved by the second of their two dicer proteins, Dcr-2 [15,17,18], which acts in concurrence with the dsRNA binding protein R2D2 to cleave the dsRNA molecules into (virus-derived) small interfering RNAs (siRNAs, or viRNAs) of mostly 21 nucleotides (nts) in length [19]. The double stranded viRNAs are transferred to the multiprotein RNA-induced silencing complex (RISC) where the duplex is passed to a key member, Argonaute 2 (Ago-2), also called the ‘slicer’ protein [20]. This protein unwinds the viRNA duplex and incorporates one strand (the guide strand) of the digested viRNA molecule while the endonuclease, C3PO, removes the unnecessary passenger strand resulting in the activation of RISC for effective target silencing [21]. Ago-2 is able to use the viRNA guide strand to recognise homogenous sequences, such as viral single stranded RNA, and this results in sequence specific degradation and silencing of that particular genetic sequence (Figure 1A) [22]. In this way RNAi suppresses viral replication. Orthologues of Dcr-2, Ago-2 and R2D2 have been shown in several vector species including *Aedes aegypti*, *Anopheles gambiae *and* Culex pipiens *[23,24,25]*.* Early experiments revealed that inhibition of flaviviruses and alphaviruses in cultured *Ae. aegypti*, *A. gambiae *and *Ae. albopictus* mosquito cells (reviewed by [14]), as well as whole *Ae. aegypti* mosquitoes, can result from the transient expression or introduction of long viral dsRNA into the cell cytoplasm, proving the existence of an exogenous RNAi pathway. These observations soon led to the first description of RNAi as an antiviral mechanism controlling the replication of o’nyong-nyong virus (ONNV, *Togaviridae*) in anopheline mosquitoes [26]. Subsequently, it was shown that Dcr-2, Ago-2, R2D2 and, to a lesser extent, Ago-3, were key enzymes involved in the antiviral response against a number of alphaviruses and flaviviruses, similar to observations in drosophila [26,27,28]. 

Several frequently used cell lines have proven to be good models for antiviral RNAi responses, mainly through work with alphaviruses and flaviviruses. *Ae. aegypti-*derived Aag2 and *Ae. albopictus-*derived U4.4 cell lines are known to have functional antiviral RNAi responses [29,30]. Recent work has shown that *Ae. albopictus* derived C6/36 and C7/10 cells are particularly permissive for a number of arboviruses, allowing for efficient viral replication and is hypothesized to be as a result of encoding defective Dcr-2 proteins. This further implicates Dcr-2 in the stimulation of a successful antiviral RNAi response [29,31,32]. In comparison to the Dcr-2 proteins expressed by U4.4 cells, which have been shown to contain DExH/D-box protein family and helicase domains followed by a domain of unknown function (DUF), a PAZ domain and two RNase III domains, C7-10 cells include a 33 amino acid in frame deletion between the DUF and PAZ domains [29]. The Dcr-2 encoded by C6/36 cells has been demonstrated to contain a homozygous frameshift mutation as a result of a single nucleotide deletion that generates a downstream nonsense codon in the same reading frame. The supposed truncated protein is therefore missing part of the PAZ domain and both RNase III domains. These mutations have resulted in the Dcr-2 null phenotype and so these cell line models provide valuable, well controlled study conditions for the RNAi pathway and possibly others. Their importance is likely to increase in the future, in particular for biochemical studies due to the ease with which they can be handled compared to live mosquitoes.

For arboviruses, such as negative-strand RNA viruses (in particular bunyaviruses, as well as some rhabdoviruses) surprisingly little information is available as a result of immunity studies in exogenous RNAi-competent cells focusing on drosophila or drosophila derived cells [31,33]. Future work in mosquitoes or mosquito derived cell lines is required to establish whether findings from drosophila can be extrapolated to a more natural host system. Moreover, little is known about this antiviral pathway in key arbovirus vectors such as midges, which transmit BTV (*Reoviridae*) and possibly SBV (*Bunyaviridae*). Undoubtedly, future research will have to look at these less studied arboviruses and vectors.

The exogenous antiviral RNAi response intervenes at key stages during arbovirus dissemination into and out of the mosquito vector. It is known that, at least for Sindbis virus (SINV, *Togaviridae*), the exogenous RNAi pathways in the midgut acts as an escape barrier and, in the case of DENV (*Flaviviridae*) infection, it limits virus titres in mosquito saliva [28,34]. Further research into this branch of the antiviral small RNA pathway may provide useful targets for the prevention of arboviral transmission by arthropod vectors and may give important clues regarding vector competence and transmission [28].

## 3. Characteristics and Biological Roles of viRNAs in the Exogenous siRNA Pathway

The nature and origin of viRNAs in arbovirus-infected mosquito cells has been an area of intense research which was revolutionized by high-throughput sequencing technology. In drosophila virus models, viRNAs were found to be mainly 21 nt in length and most likely derived from replication intermediates [35,36]. Deep sequencing technologies have also allowed important insights into virus-derived small RNAs from arboviruses and we shall attempt to summarize recent findings in this field. Infections with alphaviruses (of the *Togaviridae *family) SINV, Semliki Forest virus (SFV), ONNV and CHIKV of mosquito cell lines and/or whole mosquitoes also resulted in the generation of viRNAs that are largely 21 nt in length. Interestingly, the viRNAs generally only made up a minor percentage of the total RNA population [27,29,30,32,37,38]. Similarly for infections with West Nile virus (WNV, *Flaviviridae*) and DENV (*Flaviviridae*) this was also shown to be the case [32,39] as infection again resulted in the production of 21 nt viRNAs in RNAi competent mosquitoes/cells which is expected for viRNAs generated by Dcr-2 in the exogenous siRNA pathway. However, DENV infection of C6/36 cells generated viRNAs of 27 nt, consistent with an alternative cleavage mechanism [32]. 

A key feature of viRNAs produced during arbovirus infection is their asymmetrical distribution across the entire length of both the genome and antigenome with apparently random generation patterns of viRNA, high production ‘hot spot’ and no/low production ‘cold spot’ regions [29,30,31,32,37,38,39,40]. The reasons for this are unclear, however; it is unlikely that this is due to the processes of virus replication itself (Donald *et al.*, in preparation). It has also been shown in drosophila that abundant viRNAs produced during flock house virus (FHV, *Nodaviridae*) infection are not integrated into RISC and do not mediate the antiviral response [35]. These experiments have not yet been carried out in mosquito cells and this could prove important to better understand the fate of viRNAs following Dcr-2 cleavage. It has been shown during SFV infection of mosquito cells that there is no correlation between the sequence structure and the frequency or location of viRNAs produced [30]. This implicates dsRNA-replication intermediates as the substrate of Dcr-2 and initiator of RNAi rather than secondary structures in viral genomes. This has also been suggested for viRNAs from DENV-infected Aag2 cells [32], and again the reasons for the distribution patterns of viRNAs are not clear yet.

## 4. Viral Evasion or Antagonism of RNAi

Interestingly, hot spot viRNAs have been shown to be less active mediators of antiviral RNAi compared to cold spot viRNAs, and it has been suggested that this may prove to be an evasion or decoy strategy in the absence of a viral protein antagonizing RNAi [30]. Indeed, engineering potent RNAi inhibitors into alphavirus genomes results in enhanced lethality for the mosquito vector [38,41], and it is possible that a weaker evasion or decoy strategy may strike the right balance between vector survival and virus replication. This resembles findings obtained with FHV-infected *D. melanogaster*, as discussed above, where abundant viRNAs were found to be poor mediators of RNAi and a high percentage were not integrated into RISC [19]. Such decoy strategies may not be unique to insects. In plants, it has been shown that *Arabidopsis thaliana* produces 21, 22 and 24 nt viRNAs following infection by Cauliflower mosaic pararetrovirus [42]. Analysis of the origin of these viRNAs indicates that they originate from dsRNA predominately produced from both polarities of a 600 bp non-coding leader region. They are generated by all four of the dicer-like (DCL) proteins present in plants however, despite a reduction of viRNAs in quadruple DCL knockout plants, there is no inhibition in viral replication and a viRNA decoy strategy has been suggested.

Despite antiviral exogenous RNAi responses, mosquitoes are unable to clear the infection and arboviruses disseminate to the salivary glands. This suggests that they are able to evade or antagonize the RNAi response in some way [28,43]. It has been hypothesized that arboviruses may also be able to circumvent the RNAi response by hiding their dsRNA molecules in replication complexes as has been shown for positive-strand RNA viruses in mammalian cells [44,45]. Positive-strand RNA viruses, which include key arboviruses of the *Flaviviridae* and *Togaviridae* families, create cytoplasmic vesicles produced from mammalian host cell membranes which enclose the viral replication complexes [27,28,46,47]. By sequestering their replication-associated dsRNA, arboviruses of some families may be able to hide from or delay the RNAi response, but there is currently no experimental proof of this in mosquito systems. However, a strain of SINV producing more viRNAs was shown to infect mosquitoes less efficiently when compared to highly infective SINV [27] and clearly more work is required to understand the mechanisms behind these observations. Future work will therefore have to answer questions on when and where antiviral RNAi responses are initiated.

It can therefore be suggested that arboviruses do use RNAi-antagonistic strategies but that these take a different form to the classical RNAi inhibitor proteins known for many pathogenic insect viruses [48,49]. Active inhibition of RNAi is commonly used by “proper” insect viruses that express virus-encoded suppressors of RNAi (VSRs) which interact directly with components of the RNAi response [15,17]. A common mechanism of inhibition is that the suppressor protein will bind to and sequester viral dsRNA molecules. Examples include HC-Pro in potyviruses (*Potyviridae*), p19 in tombusviruses (*Tombusviridae*) and B2 in FHV and Wuhan nodavirus (reviewed by [50,51,52,53]). HC-Pro, p19 and p21 suppressor proteins selectively bind to siRNA duplexes [54,55] and B2 proteins are able to bind both short and long dsRNA [51,52,53]. The expression of VSRs in the context of arbovirus infection has led to recent ground-breaking discoveries. If expressed by mosquito-borne recombinant alphaviruses, VSRs such as nodavirus B2 proteins are highly potent and capable of enhancing the synthesis of viral RNA whilst reducing viRNA production and causing diminished survival rates in infected mosquitoes [38,41]. Similarly, expression of p19 by SFV greatly enhances virus spread and replication in mosquito cell culture [56]. It can therefore be suggested that the expression of VSRs in arboviruses has been selected against to allow an appropriate balance between vector survival and viral transmission to be formed. 

It is possible that flaviviruses do encode a VSR but that it has weak activity. Flaviviruses specifically produce subgenomic flavivirus RNA (sfRNA) molecules which have comparable structure to the virus associated (VA) RNA molecules encoded by human adenovirus type 5 (Ad5). These act as VSRs in mammalian cells through interacting with Dicer as competitive substrates. They are then cleaved and loaded into RISC causing inhibition of viral dsRNA processing [57]. It will be interesting to see if similar effects take place in mosquitoes and if more RNA-based decoy or inhibition strategies are described.

## 5. Non-Cell Autonomous RNAi and Cell-to-Cell Spread of Small RNAs: Another Layer of Complexity to Exogenous RNAi

In plants, systemic RNAi, *i.e.*, the spread of siRNAs to surrounding uninfected tissues (cell to cell or through plant vasculature), has been shown to be crucial to antiviral responses [58] and a recent study in drosophila has demonstrated that a systemic aspect is also crucial in *D. melanogaster* antiviral defenses [59]. Similar processes have been described in arbovirus-infected mosquito cells where SFV has been shown to be unable to prevent the spread of viRNAs to neighbouring *Ae. albopictus*-derived U4.4 cells [56]. The viRNAs are distributed from a single, autonomously infected cell to adjoining cells (requiring cell contact) and this successfully limits infection. These studies suggest that systemic/non-cell autonomous aspects of RNAi are more widespread than previously thought. Non-cell autonomous RNAi has been described in vertebrates and research in this area may become more prominent in the future [60]. In nematodes and fungi viRNAs are amplified by RNA dependent RNA polymerases (RdRps) through *de novo* dsRNA synthesis intensifying the signal [61]. In plants, systemic RNAi also relies on this amplification of small RNAs but, with the exception of ticks [62], this has not been described in arthropods. Further investigations into RdRp activities in arthropods are necessary but short distance spread may not require amplification to mediate antiviral activity.

## 6. The Endogenous siRNA Pathway

Endogenous siRNAs (endo-siRNAs or esi-RNA) have been found in several organisms, including *Caenorhabditis elegans*, *D. melanogaster* and mammals. Their production can either be dependent on the host-encoded RdRp, as in the case of *C. elegans* [63,64] or independent, as shown for mammals [65,66] and *D. melanogaster* [67,68,69,70,71,72]. Just like viRNAs, endo-siRNAs are dsRNA molecules with a length of 21 nt however, they derive from sense-antisense RNA double strands transcribed from transposons, structured transcripts folding into stem loop structures (distinguished from miRNA precursors by their extended stem length) or overlapping transcripts of protein-coding genes and unannotated regions. The *D. melanogaster* exogenous and endogenous siRNA pathways share several key proteins and features such as cleavage from long dsRNA precursor by Dcr-2, the involvement of dsRNA binding protein R2D2 and the incorporation of siRNA molecules into the so-called siRISC with Ago-2 as the catalytic compound [68,69,70] (Figure 1A, B). However it has been shown that the loquacious (Loq)-PD isoform interacts only with Dcr-2 in the endogenous siRNA pathway [73]. In addition, endo-siRNAs often have sequence substitutions, mostly A to G (~20% in Ago-2 associated endo-siRNA in *D. melanogaster *derived Schneider-2 cells), likely due to ADAR editing [70,74]. This in turn would suggest that the double stranded endo-siRNA precursor is already present in the nucleus, as ADAR is strictly nuclear and edits only dsRNA molecules [74], in contrast to the exogenous siRNA pathway that is entirely cytoplasmic. Besides, endo-siRNA precursors often have natural mismatches and bulges, unlike substrates for the exogenous siRNA pathway.

Nothing is presently known about proteins involved in the endo-siRNA pathway in mosquitoes, but it is expected to be similar to *D. melanogaster*. It is believed that endo-siRNAs, which are derived from transposons, are used to repress transposons in soma and germline cells to ensure genome stability [65,67,68,69,70,75]. This is partly supported by the observation that 18–27% and 28% of endo-siRNA molecules produced in *D. melanogaster* or *Ae. aegypti* respectively are shown to map perfectly to known transposons in these organisms [68,76]. Deep sequencing revealed no hot spots of endo-siRNA production but instead there was an even distribution throughout the whole genome mapping to transposons [65,66,75,77]. Little is known about the function of endo-siRNA molecules targeting non-transposon regions, the effect of mutations or lack of the endo-siRNA pathway in invertebrates. It has been suggested that endo-siRNAs can also repress a variety of protein-coding genes in addition to transposons, which could explain the observation that endo-siRNAs are involved in maintaining resistance to temperature fluctuations, heterochromatin formation, energy homeostasis and protection against stress and ageing in drosophila [78,79,80]. Nothing is known about an antiviral activity of endo-siRNA molecules in either mammals or invertebrates. VSRs such as drosophila C virus (DCV, *Dicistroviridae*) 1A protein and FHV B2 protein have been shown to interfere with the endo-siRNA pathway in transgenic flies, resulting in higher expression of certain transposon transcripts [81]. More research is needed to investigate if this also occurs during virus infection and the effect it has on viral replication. Recently, an induction in mRNA derived endo-siRNAs has been reported in *Ae. aegypti* upon infection with SINV [82]. Future research is required to show the effect the induced mRNA-derived endo-siRNA has on the viral infection and if such induction could also be observed for other viruses. 

Sequences derived from rhabdovirus [83] and flavivirus [84] genomes have been found to be incorporated into mosquito genomes. It is believed that these sequences have arrived by multiple independent integrations [83]. At the moment, it is not yet known if they are transcriptionally active or if small RNAs are produced from these sequences. If this is the case, it is tempting to speculate that these small RNAs, targeting the incorporated genome sequences, could result in the mosquito acquiring natural resistance to other viruses sharing sequence homologies. This may define or at least influence the vector competence of mosquitoes for some arboviruses. Similar “co-protection” has been recently described for *C. pipiens* mosquitoes persistently infected with Culex flavivirus (*Flaviviridae*), an insect specific flavivirus, on vector competence for WNV [85]. It is not known if this observed “co-protection” is due to the production of small RNA molecules and the RNA silencing response or other factors like nutrient depletion. More research is needed to answer this question

## 7. The microRNA Pathway

The miRNA pathway is a gene expression regulation mechanism shared by many organisms (plants, mammals and invertebrates), to mostly down-regulate genes although some positive regulations have also been reported [86,87,88,89,90,91]. 

This pathway shares some similarities with the siRNA pathways. It also starts with cleavage of the dsRNA inducer molecules into small dsRNA molecules, followed by incorporation of the guide strand into RISC (often denoted miRISC) and targeting complementary single-stranded RNA molecules (Figure 1C). Differences between the siRNA and miRNA pathway are in the effector proteins and location. The siRNA pathways are mostly cytoplasmic while the miRNA pathway has both nuclear and cytoplasmic phases. In animals, chromosomal miRNA clusters are mostly RNA polymerase II transcripts that fold back into a partial dsRNA stem-loop structure molecules called primary miRNA (pri-miRNA). In most cases, the nuclease Drosha (in co-ordination with Pasha, or DGCR8 in mammals) cleaves the pri-miRNA molecule into a precursor miRNA (pre-miRNA) of ~70 nt, which is exported from the nucleus. Some miRNAs (so-called mirtons) can be generated by introns without the need for Drosha, simply by using the splicing machinery [92]. Cleavage of the pre-miRNA by Dicer-1 in co-ordination with loq (TRBP in mammals) in the cytoplasm generates the mature 21/22 nt miRNA/miRNA* duplex molecules which, unlike siRNAs, are not completely double stranded (reviewed by [86]). After incorporation and unwinding of the miRNA/miRNA* duplex, the miRNA guide strand is kept within miRISC and the miRNA* strand is mostly degraded [93] but can occasionally become incorporated [90,94]. In drosophila, Ago-1 is part of miRISC instead of Ago-2, as in the siRNA pathway. The miRISC use the guide strand to find either perfectly or partly complementary RNA sequences, resulting in degradation, translational inhibition or both. The exact pathways defining either translation inhibition or target degradation are not known. It is believed that high complementarity between a miRNA and its target RNA results in degradation [95,96,97]. For animal miRNA molecules, perfect binding of the seed region (2–8 nt of the miRNA 5` terminus) is important and sufficient for miRNA function. In comparison, plant miRNAs usually map perfectly along the entire length to their target RNA [91,98]. Therefore, it is not surprising that one miRNA molecule can potentially regulate a variety of genes. In addition, host-encoded miRNA molecules can target transcription factors that are also in charge of regulating multiple genes [99]. Knockout experiments in *D. melanogaster* and *Bombyx mori* indicate the involvement of miRNAs in cardiogenesis, neurogenesis, muscle growth, stress resistance, fat metabolism, proliferation and development [100,101,102,103,104,105]. The miRNA molecule expression is tightly regulated and often differs per tissue and during the developmental stages.

Little is known about the miRNA pathway in mosquitoes, although it is believed to use the same proteins as *D. melanogaster*. Bioinformatic approaches of the sequenced mosquito genomes have shown that *Ae. aegypti*, *C. pipiens* and *A. gambiae* encode putative genes for the miRNA pathway, such as Dicer-1, Drosha, Pasha, loq and Ago-1. Two putative genes for Ago-1 have been identified in the *Ae. aegypti* genome [23,24,106,107]. In addition, experiments in *A. gambiae* or derived cell lines have shown the presence of Ago-1 transcripts [26,108]. Deep sequencing and northern blot analysis have determined the expression of several miRNA molecules in different mosquito species and their derived cell lines: *C. quinquefasciatus* [109], *A. gambiae*, *A. darlingi* [110] and *Ae. aegypti* [111] suggesting the presence of a functional miRNA pathway in mosquitoes. Some of the identified miRNA molecules are mosquito-specific while others are conserved between arthropods and mammals [109,110,111]. 

Although limited research has been performed on the interaction of the miRNA pathway and viruses, accumulating evidence indicates an important role for miRNA molecules in the antiviral response in mammals. This can either be by directly targeting viral transcripts or by altering the expression of host transcripts that are important for viral replication (reviewed by [112]). In addition, several mammalian infecting viruses (mostly DNA viruses, belonging to the herpes family) encode miRNA molecules of their own to regulate host and/or viral transcripts (reviewed by [113]). Although the miRNA pathway has been investigated in great detail in mammals, less information is available about miRNAs and virus-host interaction in invertebrates.

Transgenic *D. melanogaster* expressing VSRs had no alteration in the miRNA profile [81], in contrast to previous experiments in transgenic plants [114]. However, differential expression of miRNAs has been observed in WNV-infected *C. quinquefasciatus* mosquitoes [109] and *Helicoverpa zea* fat body cells infected with *Heliothis virescens* ascovirus (HvAV-3e, *Ascoviridae*) [115]. Alterations in miRNA patterns can be due to either intrinsic or extrinsic factors. Possible intrinsic factors could be host receptors which sense viral infection and result in signal cascade activation. Extrinsic factors could be virus-encoded small RNA molecules or proteins, such as VSRs interacting with the host pathway and thereby facilitating viral replication, inducing persistence and/or interference with the host defense response. Direct interaction between a host encoded miRNA molecule in *Helicoverpa zea* fat body cells and HvAV-3e has been reported [115,116]. Several other direct interactions between host miRNAs and viruses have been predicted but not yet experimentally confirmed. 

Most arboviruses are RNA viruses and it has been argued that RNA viruses do not encode miRNA molecules, as they lack the nuclear phase and the miRNA molecule would target its own genome. However, Shapiro and colleagues have recently shown that insertion/engineering of the mmu-miRNA124-2 into SINV genome had no negative effects on viral replication or viral titer in mammalian cells. In addition, it was shown that a functional miRNA molecule was produced from the inserted miRNA sequence by a Dcr-1-dependent but DGCR8 independent process, indicating a non-canonical miRNA production pathway like the previously described mirtons [117]. Similar results were observed for tick-borne encephalitis virus (TBEV, *Flaviviridae*) expressing the miRNA-BART2 hairpin precursor from the herpesvirus Epstein-Barr Virus (EBV, *Herpesviridae*) [118]. Recently, an arbovirus (Kunjin virus, *Flaviviridae*) was shown to encode a miRNA molecule in its 3`UTR (kun-miRNA1). This miRNA-1 positively regulates the host encoded GATA4 transcript, which is required for efficient viral replication in mosquito cells. Expression of the kun-miRNA1 molecule was detected in several mosquito cell lines but not in mammalian cells [89]. This is in line with earlier observations showing manipulation of the miRNA pathway to be often specific to a pathogen/host interaction [119,120]. 

## 8. Piwi-Interacting Pathway

Piwi-interacting RNA (piRNA) molecules are 24–32 nt in length and interact with proteins of the PIWI subfamily (PIWI, Aubergine [Aub] and Argonaute-3 [Ago-3]) that are predominantly expressed in germline cells of a variety of organisms (e.g., mouse, zebrafish and drosophila) [121,122,123,124,125] (Figure 1D). Although their induction pathway is still not fully understood it appears to be Dicer-independent [126]. Primary piRNAs are probably derived from long single-strand precursor RNAs that are transcribed in antisense from genomic regions with defective transposons. These primary piRNAs target/interact with transposon-derived sense RNAs, resulting in cleavage of the RNA and giving rise to secondary piRNA molecules [121,122,123,124,125]. These secondary piRNA molecules are integrated into Ago-3 and are used as a guide to find complementary antisense RNA that is then cleaved into antisense primary piRNAs [121,122,123,124,125]. Primary and secondary piRNA molecules have specific features due to this so-called “ping-pong” amplification mechanism. Primary piRNAs are mostly associated with Aub and PIWI proteins and have a bias for uridine as their first nucleotide in 5’. Their first ten nucleotides are often complementary to the Ago-3-bound secondary piRNA molecules. Secondary piRNAs are mostly in sense orientation and have a bias for adenine at the tenth position [121,122,123,124,125,127]. 

In *D. melanogaster*, different sizes of piRNAs (25 nt, 24 nt and 23 nt) have been associated with PIWI, Aub and Ago-3. In drosophila, Aub and Ago-3 have been exclusively found in germ cells, compared to PIWI which is also expressed in follicular cells (of somatic origin) surrounding the germ cells [128]. Knockdown experiments in *D. melanogaster* showed that silencing of PIWI/Aub or Ago-3 leads to less piRNA production and a lack of transposable element silencing, however, the endo-siRNAs pathway was at least partly able to take over [127]. Aedine and culicine mosquito species have been found to have an expanded number of piRNA pathway related proteins, encoding six or seven PIWI proteins, instead of the two as found in *D. melanogaster* and *A. gambiae *[23]. On the other hand, no Aub protein has been identified in aedine, culicine or anopheline species, although each has shown to express one Ago-3 protein [23,24,106,107]. Recent research suggests differences in the piRNA pathway in *Ae. aegypti* and *D. melanogaster*. Only a small amount of piRNAs produced in *Ae. aegypti* map to transposons, compared to *D. melanogaster* where the opposite is observed. In fact, most *Ae. aegypti* piRNAs map to coding regions and in some instances even to virus-derived regions in the genome [76]. Comparing the transposon load of the genomes of drosophilid, anopheline and aedine species shows that the *Aedes* genome encodes for more retro-transposons, ~15%, 16% and 47%, respectively [23,24,76,129,130]. For *D. melanogaster*, transgenic lines have been established with transposon-based technology, but this was rather inefficient in *Aedes* due to fast inactivation of the transduced genes [131,132]. For example, transgenic *Ae. aegypti *mosquitoes with an inverted repeat against part of the DENV sequence incorporated in their genome showed strong resistance against DENV infection for the first generations [133]. Eventually DENV resistance weakened and was lost, and this corresponded to a lack of expression of the incorporated DENV sequence although no mutation could be found in the incorporated sequence [134]. 

Several recent reports have suggested antiviral activity of piRNAs in *D. melanogaster* as well as in aedine mosquitoes and derived cell lines. Deep sequencing of drosophila ovary somatic sheet (OSS) [36] cell lines showed high levels of piRNAs against DCV and American nodavirus (*Nodaviridae*). The piRNAs mapping to other viruses were also detected but at lower concentration. Most of these viral piRNAs were in sense orientation and derived from positive-strand RNA and dsRNA viruses, suggesting viral mRNA and genome as the source. In addition, these piRNAs had a strong bias for uridine at position one and no preference for adenine at position ten, resembling primary piRNA features. As the OSS cells express PIWI but no Aub or Ago-3 they would be expected to have no functional primary (PIWI and Aub dependent) nor secondary (expected to be Ago-3 and Aub dependent) piRNA pathway. The observation of viral specific piRNAs indicates a non-canonical piRNA pathway that depends only on PIWI. In addition, knockdown of PIWI in drosophila, results in a higher WNV production comparable to what is observed in Ago-2 mutants [135]. The role of viral piRNAs in controlling arbovirus infection in mosquitoes is not clear. Knockdown experiments of Ago-3 in *A. gambiae* showed an increase in virus production [26], and this supports the piRNA pathway possessing antiviral activity, at least in this mosquito species. Similar affects in other mosquito species are yet to be observed. The fact that PIWI and Ago-3 transcripts could be detected in somatic cells of *Ae. albopictus* (head and thorax) [29] and *Ae. aegypti*-derived Aag2 cells [136] indicates a possible difference in the piRNA pathway between mosquitoes and drosophila, where they are thought to be restricted to the germline or follicle cells. Although the exact origin of Aag2 cells is not clear, the lack of expression of the *Nanos* transcript indicates a non-germline origin [136]. However, recent detection of piRNA-like RNA molecules interacting with PIWI-like proteins in the somatic tissues of mouse, drosophila and macaques [137] suggests that the piRNA pathway could also have an additional role in these organisms. Until recently, the production of 24–29 nt viral specific RNA molecules was only in cells with a deficient siRNA pathway, like the *Ae. albopictus*-derived C6/36 or C7/10 cells [29,31,32]. 

Initial reports indicating the involvement of piRNA or similar pathway in the antiviral response in mosquitoes show the production of 24–30 nt DENV-specific RNA molecules in infected *Ae. aegypti* mosquitoes. Although most of these piRNAs were in sense orientation, as reported for drosophila, they mostly lacked the specific U_1 _bias. In contrast, SINV–derived piRNAs had a preference for uridine at the 5` end in *Ae. aegypti* [40]. Similar results were recently observed by Morazzani and colleagues [29], who analyzed infection of *Ae. albopictus* and derived cell lines (U4.4, C6/36 and C7/10) and *Ae. aegypti* with CHIKV. They observed that in all of these cell lines and mosquitoes, viral specific small RNAs of 24–30 nt in length were produced, representing features of the ping-pong based piRNA production pathways. However, in whole mosquitoes and U4.4 cells, which have a functional Dcr-2 dependent antiviral siRNA pathway, most viral specific small RNAs were 21 nt in length and are thought to be produced by Dcr-2. On the other hand, in Dcr-2 deficient C6/36 and C7/10 cells, most viral specific small RNAs were 24–30 nt in length and displayed ping-pong features [29]. The production of SINV-derived piRNAs in the range of 24–30 nt with a peak at 27–28 nt in U4.4 and C6/36 cells shows that the production of viral specific piRNAs is not specific for CHIKV infection [136]. In addition to the U_1_ bias, an A_10_ bias in antisense small RNAs was found, indicating involvement of a ping-pong mechanism, as described earlier. The observation that similar molecules were produced in C6/36 cells infected with the segmented negative strand RNA bunyavirus La Crosse (*Bunyaviridae*) [136] indicates that the production of virus-specific piRNAs in mosquitoes by the ping pong mechanism can affect several types of arboviruses. However, the low amount of 24–30 nt viral specific small RNAs in C6/36 infected with WNV may point towards possible differences in how the piRNA-based RNA silencing response affects viruses of different families [31]. It is not yet known how viral piRNA production is induced in mosquitoes. Experiments with transgenic CHIKV expressing Nodamura virus B2 or FHV B2 points to viral dsRNA as a precursor substrate in the biogenesis of viral piRNAs which would again indicate a non-canonical piRNA pathway [29]. The dsRNA inducer molecule could be either long dsRNA, as described for the exogenous siRNA pathway or possibly viRNAs. Another possibility could be that some of the proteins present in the RNAi pathway (e.g., Ago or Dicer) start a signal cascade resulting in the production of viral specific piRNAs. In drosophila it has been reported that Dicer is not only able to act in the RNAi pathway but can also induce another antiviral cascade, resulting in the expression of antiviral proteins [138]. Therefore, it would be interesting to investigate how viRNA and piRNA production would differ for a virus expressing a protein that does not interfere with the activity of Dcr-2 by binding long dsRNA, such as tombusvirus p19. The importance of arbovirus-derived piRNA-like small RNAs in the host defense against viruses is at present not fully understood. Perhaps other small RNA pathways, in addition to the exogenous siRNA pathway can add to antiviral defenses, as Dcr-2 deficient cell lines are affected by arbovirus infection [29]. More research is needed to really understand the potential role of the piRNA and its proposed antiviral activity in mosquitoes. 

## 9. Concluding Remarks

The role of small RNA pathways in controlling arbovirus infection in mosquitoes is now widely recognised but the regulation of these pathways and the biochemical processes underlying antiviral activities are still largely unknown. More genetic tools to manipulate mosquitoes and mosquito cell lines will be required to further investigate this but, at least for some pathways, we are beginning to understand their importance. This area of investigation will undoubtedly set the pace in understanding arbovirus/vector interaction for the foreseeable future.
